# The Challenge of Generating Sustainable Value: Narratives About Sustainability in the Italian Tourism Sector

**DOI:** 10.3389/fpsyg.2020.577612

**Published:** 2020-12-23

**Authors:** Laura Galuppo, Paolo Anselmi, Ilaria De Paoli

**Affiliations:** Department of Psychology, Università Cattolica del Sacro Cuore, Milan, Italy

**Keywords:** sustainability, tourism, narrative research, stakeholder, environment

## Abstract

Tourism is capable of distributing wealth and participating substantially in the economic development of many countries. However, to ensure these benefits, the planning, management, and monitoring of a sustainable offer become crucial. Despite the increasingly widespread attention to sustainability in this sector, however, the concept of sustainable tourism still appears fragmented and fuzzy. The theoretical frameworks used in many studies often reduce sustainability to its environmental or social aspects and consider such pillars as separate issues. Furthermore, although most studies acknowledge that a potentially wide number of stakeholders play a role in sustainable tourism production, they have so far focused on host communities, tourism producers, or tourists themselves independently. Fewer explorations have addressed simultaneously different stakeholders, their perceptions of sustainable tourism experience, and the various concerns and tensions that may arise. This study aims to investigate sustainability issues in tourism by considering the voices of two relevant stakeholders involved in “co-producing” the tourism experience: tourists and tour operators. Based on a qualitative study conducted in Italy, the article critically discusses how travelers and tour operators craft the sustainability idea, the implicit assumptions that rely on their different perspectives, and their practical implications. The results highlight four different narratives on sustainable tourism, which are related to different assumptions on sustainability and actions legitimated to generate sustainable value. Finally, the article offers insights into how to develop a more holistic and critical approach to sustainable tourism through education and communication.

## Introduction

Over the past few decades, tourism has grown continuously and now represents 10% of global employment and 10% of global gross domestic product (GDP). With the number of domestic and international arrivals estimated to reach 15.6 billion and 1.8 billion by 2030, respectively, tourism is expected to continue generating significant benefits in terms of both socio-economic development and job creation worldwide (World Tourism Organization and International Transport Forum, 2019). In Italy, tourism contributes significantly to the economy. In 2019, it accounted for 13% of GDP through direct and indirect effects. In 2018, the tourism industry employed 2 million people, accounting for 8% of total employment. Travel exports represented about 40% of total service exports in 2018 (Organization for the Economic Cooperation and Development [OECD], 2020).

Therefore, tourism is capable of distributing wealth and participating substantially in the economic development of many countries (Higgins-Desbiolles, 2017). However, to ensure these benefits, the planning, management, and monitoring of a sustainable offer become increasingly crucial. The risks of tourism are many, including pollution, the deterioration of destinations, and the absence of a consolidated international regulatory system (World Tourism Organization and International Transport Forum, 2019). Tourism thus represents a unique context for studying sustainability and sustainable behaviors, both at the individual and the organizational levels.

A commonly acknowledged definition of sustainable tourism states that any form of tourism can be defined as sustainable when the current and future economic, social, and environmental effects meet the needs of visitors, the environment, and the host communities (World Tourism Organization and United Nations Environment Programme, 2019). In recent years, tourism policies and programs in Italy have focused explicitly on sustainability. As reported by Organization for the Economic Cooperation and Development [OECD] (2020), the vision is to “generate sustainability in economic, social, and cultural terms by drawing on the value of the wider tourism offer underpinned by local businesses” (p. 208). An increasing number of tourists and tour operators also appear sensitive to sustainability. In a recent Italian survey, 81% of respondents have reported knowing the definition of “sustainable tourism” as an experience that respects the environment and seeks to reduce energy and other resources. Sustainable development is finally viewed as an opportunity to increase destinations’ competitiveness and growth, according to 89% of respondents (Report Fondazione Univerde, 2019).

Despite the increasingly widespread interest in and attention to sustainability in this sector at both the Italian and international levels, the concept of sustainable tourism still appear ambiguous and fuzzy. Many research studies in this field have focused on identifying sustainability indicators to assess the effect of a tourist’s experience, while other studies have explored the profiling of so-called responsible tourists or sustainable locations, which are now becoming a very promising market segment (see, for instance, Zavattaro, 2014). However, as Caruana et al. (2014) stated, many of these studies have relied on standardized indicators that are too general and fragmented to explain the complex and situated processes that influence a sustainable tourism experience. For example, the sustainability frameworks used in many studies often reduce the concept to its environmental or social aspects and consider such pillars as separate issues. Their interconnectedness and the tensions between them remain under-explored (Smith and Lewis, 2011; Galuppo et al., 2014). For these reasons, these indicators still fight to be useful for practice (Baden-Fuller and Mangematin, 2013; Jones, 2014). Finally, although most research studies acknowledge that a potentially wide number of stakeholders play a role in sustainable tourism production, they have thus far focused on host communities, tourism producers, or tourists themselves separately. Fewer explorations have addressed different stakeholders’ voices simultaneously, paying closer attention to how stakeholders frame their experience of sustainable tourism and considering the various concerns and tensions that may arise. For these reasons, in recent years, several scholars have called for a more integrated approach to sustainability to advance the understanding of this concept and make it more useful for practice (Banerjee, 2003; Bradbury, 2003; Allen et al., 2015, 2017).

In the article, the authors adopt an interpretive and critical approach to tourism sustainability by referring to the framework proposed by Allen et al. (2015, 2017). This framework supports the exploration of how people and organizations conceptualize sustainability in their everyday talks and narratives. The authors also propose the deconstruction of how issues of sustainability are discoursively crafted, for example, by questioning the idea of a natural environment separated by people and by addressing contradictions stemming from multiple stakeholders’ voices and concerns (Galuppo et al., 2014). It is assumed that this lens might enhance the debate on tourism sustainability since it could help scholars and organizations avoid simplified views and embrace complexity with greater awareness. Therefore, the purpose of the article is to explore and deconstruct sustainability issues in the field of tourism by considering the voices of two relevant stakeholders who are always involved in “co-producing” the tourism experience: tourists and tour operators (Goodwin and Francis, 2003). Based on a qualitative study conducted in Italy, the article critically discusses how travelers and tour operators craft the sustainability idea, its implicit assumptions, and their practical implications. Finally, the article offers first insights into how to develop a more holistic and critical approach to sustainable tourism through education and communication.

## Sustainable Tourism and Sustainability: A Review of the Key Concepts

Despite the multiple elements considered in the definition of sustainable tourism, both the current practice and the research on this theme still struggle to maintain a holistic and integrated approach to it (Allen et al., 2017). The polysemy that exists around the operationalization of sustainable tourism reflects this situation. This term encompasses different coexisting meanings of tourism: ecotourism, responsible tourism, and community tourism are the most common terms. This variety is primarily attributable to the different interpretations of the value produced by sustainable tourism practices. For instance, when the concept of sustainable tourism is mainly identified with “ecotourism,” it focuses on the value of preserving the environment and existing cultural manifestations that must be admired and enjoyed in their beauty and integrity (Cobbinah, 2015). The esthetic dimension, the conservation of the environment, and the efforts to minimize tourism influence represent the main focus in this field. It might be stated that much of the discussion about sustainable tourism currently converges on this tradition. On the other hand, the so-called responsible tourism fits the spirit of the alternative tourism movement of the 1970s. According to Goodwin, responsible tourism “recognizes the importance of cultural integrity, ethics, equity, solidarity, and mutual respect placing quality of life at its core” (Goodwin, 2011, p. 16). Here the preserved value is the social well-being and cultural integrity of the local communities (Caruana et al., 2014). In this perspective, the awareness that tourism is a process that inevitably challenges consolidated balances and affects the wealth of the actors involved, which must be respected, preserved, and at the very least re-founded, takes on a central role. Finally, community-based tourism affirms an even more focused perspective on the local community, where residents are seen as the first authors and protagonists of the tourist experience and its management. In this definition, the community controls and directs the resources and goods available in a certain place based on its needs (Dangi and Jamal, 2016). Community tourism’s concerns remain highly local, as community development, community ownership, and resident control over decision-making and local benefit are prioritized (Ramkissoon, 2011; Dangi and Jamal, 2016).

The debate about which stakeholders are adept at assessing the value generated by sustainable tourism also reflects the concept’s heterogeneity and ambiguity. According to Castiglioni et al. (2019), only a few scholars have explored whether travelers or local stakeholders (destination managers, local tour operators, and small businesses) perceive sustainable tourism initiatives as such (e.g., Goodwin, 2011; Scheyvens, 2012; Passafaro et al., 2015; Loìpez-Saìnchez and Pulido-Fernaìndez, 2016). Furthermore, even when considering their representations and experiences, literature shows that stakeholder groups, for instance, the so-called responsible travelers, do not share a coherent cultural ethos, but there is significant heterogeneity in their conceptions of sustainable tourism (Caruana et al., 2014). In most cases, the awareness of the complexity and multidimensional nature of sustainability (Loìpez-Saìnchez and Pulido-Fernaìndez, 2016), which is mostly associated with environmental conservation that focuses on consumption rather than on the regeneration of the local resources, is lacking. Other studies have even reduced the concept of sustainable tourism to the mere recognition of a generally positive effect of tourism on local populations [see, for example, Gursoy et al. (2018)].

The polysemy of sustainability also involves the actions that trigger sustainability. When sustainable tourism is meant as ecotourism, the emphasis is on conservative actions and the idea of “low impact” and “safekeeping” tourism practices, which maintain the integrity of the resources and do not exhaust them. In the view of responsible tourism, the emphasis is on actions aimed at balancing between demands, needs, and resources held and exchanged by different stakeholders. From the perspective of community tourism, there is a radical shift in favor of the generation of new welfare through the promotion of active involvement of the local stakeholders (Caruana et al., 2014). Such different perspectives and approaches testify that sustainability is an umbrella term for many practices. This has several consequences for the field of tourism. First, the sustainability indicators, which refer to diverse definitions of sustainability, result to be too fragmented and, in many cases, too general to explain the complex and situated processes that influence a sustainable tourism experience (Miller et al., 2010; Caruana et al., 2014). Second, although several studies have considered ecotourism, responsible tourism, and community tourism, the dialog between these streams is limited, and the interrelation and tensions between the socio/cultural, the environmental, and also the economic “souls” of sustainability are still underexplored (Smith and Lewis, 2011; Allen et al., 2017; Galuppo et al., 2014). Third, most studies have exploited the sustainability of tourism mainly from the perspective of either the travelers or the host communities/businesses, while fewer explorations have addressed and confronted these different perspectives or discussed possible tensions and contradictions between them (McCabe and Stokoe, 2004; Mahrouse, 2011; Allen et al., 2015).

The article addresses these challenges by adopting the critical and holistic approach to sustainability proposed by Allen et al. (2017). This framework, grounded in the management learning and education field, could also be adopted to advance the knowledge and practice of tourism sustainability. Allen et al. (2017) theorize sustainability as an “embedded process” where human and non-human aspects are seen as entangled so that “the environment, communities, and people shape each other in mutually defining ways as they interact in lived experience” (p. 789). This proposition questions several taken-for-granted assumptions that affect most literature on sustainability management: the idea of a natural environment separated by people, the assumption that the sustainability pillars should be harmonically integrated with no tensions or contradictions, and the proposition that the sustainable development concept is reduced to a “(low) consumption practice”, linked to the pragmatic idea of “doing more with less.” To overcome these views, Allen et al. (2017) indicate the need to embrace a new concept of sustainability that “views humans as *attached to* rather than *detached from* nature” (p. 781), thus becoming more aware of the interrelations and eventual tensions between the *people*, the *planet*, and the *profit* pillars. This requires a different way of thinking and new reflexive attitudes. More specifically, a radical reflexive approach is invoked in people and organizations to enable them to appreciate their embeddedness and responsibility for sustainability by bringing attention to the interrelationship between their values, their actions, and their social and material world. Sustainability research should also apply a radical reflexive approach to explore how different underlying assumptions and values may shape discourses and practices of sustainability, confront eventual multiple positions and truth claims, and finally explore their practical consequences. In effect, radical reflexivity is not just about “unsettling” ways of thinking about sustainability but also about considering the relationship between a person’s view of the world and the practical outcomes of that view. Therefore, the present article aims to reflect on tourism sustainability through the lens of an “embedded” and radical reflexive approach, using bottom-up qualitative research that accounted for the voices of two relevant stakeholders—tourists and tour operators. It further aims to explore the taken-for-granted assumptions and practical implications of their sustainable tourism accounts. It is argued that this exploration might offer first insights into how to develop a more holistic and critical approach to sustainable tourism and how to promote such a view through education and communication.

## Materials and Methods

The present study aims to qualitatively explore how Italian tourists and tour operators make sense of sustainable tourism and construct their practices as “sustainable” in order to understand the meanings and value orientations that contribute to the definition of sustainable tourism and the implications of these positions. The study adopts a socially constructed perspective on reality, where sustainability is viewed as narratively constructed, mediated by people’s accounts, talks, and images (Zavattaro, 2014; Allen et al., 2017). In this work, previous qualitative studies on stakeholders’ perceptions of tourist sustainability [see, for instance, Caruana et al. (2014) and Zavattaro (2014)] have been expanded by considering the viewpoint of tour operators together with the tourists and by confronting the two “voices” to highlight positions as well as possible strategies to favor a more complex and reflexive view of sustainability. Although it should be noted that other voices, *e*.*g*., those of residents, could have been included in the study, this first exploration has been limited to travelers and tour operators as the first and the most salient stakeholders involved in co-producing a tourism experience. The relevance of their interests and effects on the destinations makes them indispensable—although not the sole—informants when dealing with sustainability.

The specific objectives of the research, therefore, have been to explore how:

- Tourists and tour operators describe sustainable tourism by focusing on the underlying beliefs, values, and attitudes related to a “sustainable tourism experience,”
- Tourists and tour operators present and justify themselves and their practices as “sustainable,”and
- “Sustainable” tourists and tour operators perceive their relationships and the challenges that they foresee concerning sustainability.

The researchers conducted 25 in-depth semi-structured interviews with 18 tourists and seven tour operators, respectively. The tourists have been selected using a purposive sampling process (snow-ball approach) based on the first screening conducted using a five-item questionnaire. The questionnaire aimed to identify people who have reported “sustainable” behaviors during their last-year travels, such as commitment to environmental conservation and local economy, responsible behaviors toward local communities, or community enhancement purposes, which in literature emerge as the core values of sustainable traveling. The screening process consisted of selecting those participants who had flagged at least three of the five “sustainable” behaviors. At the end of the screening process, 18 participants have been selected for the interviews (see Table 1 for the sample characteristics). This number has been considered sufficient for in-depth exploration, and the participants’ screening has ensured their theoretical relevance for the research questions (Silverman, 2006).

**TABLE 1:** Tourists’ characteristics (10 men and eight women).

**Name (pseudonym)**	**Age**	**Profession**	**Travel behaviors**
Carol	40–49	Business owner	Ecotourism; backpacker travels; community based tourism
Hellen	30–39	Anthropologist	Ecotourism; slow tourism; trekking
Manuel	20–29	HR Assistant	Tourism in rural areas; trekking
Linda	30–39	Architect	Tourism in rural areas: Km zero travels; trekking
Phil	50–59	Journalist	Ecotourism; trekking
Lia	50–59	Business owner	Slow tourism; charity programs
Julia	20–29	Account	Trekking; charity programs
Luka	30–39	Free lance	Ecotourism; trekking
Ann	40–49	Designer	Charity programs
Mike	60–69	Business owner	Trekking; Ecotourism
Luise	60–69	Retired	Slow tourism; eco tourism
Sofia	50–59	Housewife	Tourism in rural areas
Stephen	60–69	Architect	Community based tourism
Mario	40–49	Physician	Charity programs
Marc	30–39	Account	Eco tourism; trekking
Bill	40–49	Business owner	Km zero travels; tourism in rural areas
Tom	20–29	Teacher	Backpacker travel
Frank	30–39	Business owner	Tourism in rural areas; trekking

The tour operators were selected using a purposive sampling process. The sample has been identified from an initial population of “sustainable tourist operators,” selected from several rankings and lists of Italian travel agencies (cf. AITR—Italian Association of Responsible Tourism website) (see Table 2).

**TABLE 2:** List of tour operators.

**Name (pseudonym)**	**Affiliation**
Alex	Walden Viaggi a Piedi
Ron	ICIEI
Luca	WWF Travel
Ann	Girolibero
Rose	Oikos Onlus
Paula	Viaggi e Miraggi
Walter	AITR

The semi-structured interviews consisted of a series of open-ended narrative questions assessing a few main thematic areas: participants’ accounts of “sustainable” travel experiences with the specification of destinations, meaningful facts/events that occurred, and actors encountered; perceived learning, achievements, and criticalities related to these experiences; and desires and expectations for the future. The interviews lasted between 50 and 90 min and were recorded and transcribed verbatim. The participants were assured of their anonymity *via* the use of pseudonyms.

An interpretive thematic approach inspired by a narrative methodology has been adopted for the analysis (Ripamonti and Galuppo, 2016). The interviews have been thus considered and read as a set of narratives, as a means through which participants made sense of their experiences (Brown et al., 2008) and positioned themselves by justifying their behaviors and power (Cunliffe et al., 2004). The analysis has focused on the structure and the content of the narratives. The meaning of what was said has been explored in the content analysis, focusing on how the participants made sense of “sustainable” tourism experiences and practices. The structural analysis has examined how the participants constructed their stories, positioned themselves in them, and interpreted their relationships. The analysis has identified four main narratives, which are described in detail in the following paragraph. As Koning and Waistell (2012) state, the interpretation of the narratives described in the article is just one of the countless possible readings of the empirical material and does not intend to be incontrovertible but rather open to further analyses and reflections by the readers.

## Results: Narratives of Sustainable Tourism

**FIGURE 1:**
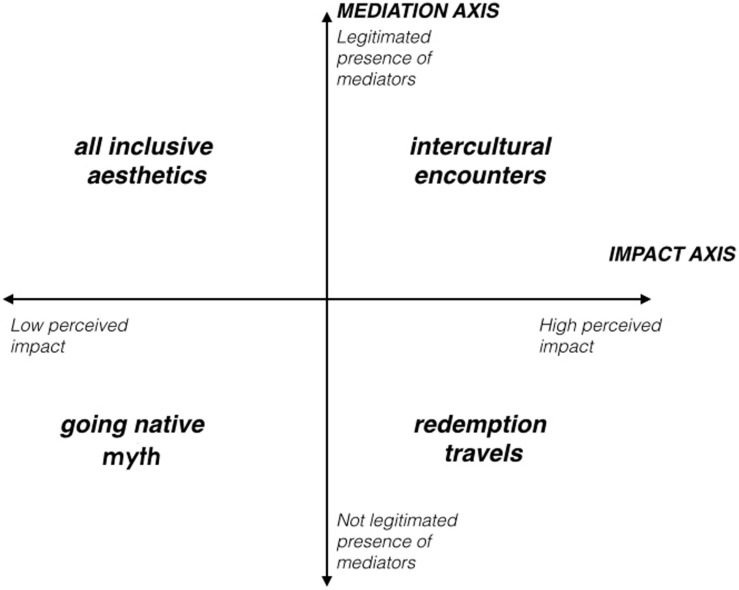
Tourism sustainability narratives.

The tourists’ and tour operators’ accounts have revealed different ideas and positions of tourism sustainability, articulated into four coherent narratives, i.e., clusters of experiences of and attitudes toward sustainable tourism. As shown in Figure 1, the four narratives are organized along two conceptual axes: (1) the perceived effect of sustainable tourism on the natural, cultural, and economic environment (horizontal axis, oriented from left to right) and (2) the perceived trust and reciprocal involvement of the two main actors—tourists and tour operators—in crafting a sustainable tourism experience (vertical axis, oriented from bottom to top). The four narratives of sustainable tourism have been outlined from the intersection of the two axes. This does not suggest that they reflect four stable and mutually exclusive tourist or tour operator typologies. Coherent with a narrative and interpretive approach, respondent accounts of sustainable tourism might move between or within the narratives, representing four possible “positions” that can be considered when dealing with tourism sustainability.

The four main narratives have been titled the “*going native*” *myth*, *redemption travels*, the *all-inclusive esthetic*, and the *intercultural encounters.* Two of these narratives, the “*going native” myth* and the *all-inclusive esthetic*, include “low impact” stories, where tourists and tour operators describe sustainable tourism as an esthetic experience and focus on environmental conservation and an idea of “escaping” from civilization. The other two, *redemption travels* and *intercultural encounters*, are “high impact” stories, where tourists and tour operators describe experiences of tourists’ high involvement with the local communities, putting at the center their search for meaningful encounters and solidarity-oriented behaviors. The narratives also differ in the reciprocal positions of the tourists and the tour operators. In two narratives, the “*going native*” myth and *redemption travels*, tour operators and tourists differ in that tourists self-organized their travels and avoided contact with other groups of travelers, while tour operators aimed to find marketing and communication strategies to reach prospective clients. On the other hand, in the other two narratives, tourists and tour operators describe the possibilities of trusting one another and starting a dialog to achieve a satisfactory and sustainable experience.

### The “Going Native” Myth

The first narrative describes a “niche” experience in which tourists, generally alone or in small family groups, travel to explore new worlds.

*“I am looking for a place that hasn’t been counterfeited yet, not homologated. If I have to be in a place that seems identical to the one where I stay all year. what am I going to do? A place that gives me a measure of what diversity is. A journey is to discover things, people, and ideas different from mine. it’s a possibility” (TS_02)*.

These tourists present themselves as explorers who, more or less, explicitly refer to an anthropological and ethnographic vocation and who are used to make adventurous trips to distant, unexplored, and authentic places where they can experience total freedom.

*“For sure, my anthropology studies have also influenced this way of traveling: recognizing and knowing cultures, understanding why people do certain things. Minimal details intrigue me a lot. Then, I also read many books about people who have traveled. I have read many books that talked about Asia: perhaps, for this reason, I feel more culturally prepared, and I tend to go more to Asian countries. (…) I love to get involved in the place where I travel, then put myself in their shoes. […] to do something that allows me to feel closer…” (TS_04)*.

As can be read in the previous excerpt, these travels are carefully yet independently planned and prepared by avoiding other mediators at the cost of running more safety risks.

“I want to see what I want. But of course, I prepare myself, I get informed. There is also the group aspect: it is not because I do not want to be in a company. I come here once in a lifetime, and time is already short. Groups are timewasting…” (TS_08).

These experiences refer to the dimension of escaping and avoiding the mass and the homologation. The journey becomes a form of exit from the known in search of possibilities and differences to explore without disturbing and being disturbed. An empathic orientation joins the esthetic one. These travel experiences are supposed to have a minimal effect on the local environmental and cultural ecosystem.

“The planet saves itself; it does not need our help. What is important is to reduce consumption and impacts as much as possible and try to do the least possible damage…!” (TS_02).

The idea of sustainability is associated with conservation and leaving values intact and informed by a self-regulating principle according to which nature, local communities, and travelers themselves must be left in peace because they can naturally sustain themselves. In these experiences, the economic side of the journey is not mentioned, except in a negative sense, in the form of consumption of exchange that corrupts.

“We are used to talking about a sustainable economy, but when there is an economic project and the investments must return, it is never sustainable for the earth!” (TS_04).

At the center of the tourism project is a tourist with a desire for discovery. Production and consumption processes are removed from the scene. Destination management or tour operators appear only as interlocutors to avoid. For tour operators, this type of tourist represents equally a distant, unreachable target on which they do not want to rely. These tourists do not even appear as threatening because they are perceived as residual and niche prospect clients.

“These are the adventurers who have always been there. They do not call themselves tourists but travelers. They don’t feel they need mediation, and sometimes they even take some risks. We are far from this target.” (TO_1).

### Redemption Travels

This narrative describes a tourist experience that has a strong ethical orientation. The tourists in these stories view themselves as citizens of the world who are actively involved and interested in entering into a dialog and helping the host communities.

“I was interested in helping the local population, but not in a sense: ‘Oh poor people,’ ‘I’m sorry, I pity them, so I give them a hand and some money.’ No, trying to help them means getting in touch with them, sharing their experiences, and, therefore, automatically supporting them in their activities in general.” (TS_17).

Moreover, these narratives reflect a search for places and contexts not yet visited by mass tourists; however, travelers do not choose isolation, as they love to have meaningful encounters, exchanges, and dialogs with local people.

“From this point of view, I would say that tourism is divided into two forms appreciated by two different people. One is mass tourism in towns like Rimini, the great beaches where people go to be surrounded by people, both day and night. The other form is for those looking for places where there are not too many people, and there is the possibility of more meaningful encounters and talks.” (TO_07).

“In Italy, the most beautiful experiences involve some forms of community tourism, in particular, the Valle Dei Cavalieri, which is a worldwide example of the Tuscan-Emilian Apennines and which also received a prize from the World Tourism Organization last year […] It had closed everything: bars, shops, school, church, post office. They left again, and today, it is a country that has regenerated itself with many activities and strong involvement of the population.” (TO_06).

Promoting the development of local contexts is a crucial idea associated with the concept of sustainable tourism. Local communities are sometimes perceived as active participants in the exchange, sometimes as more fragile recipients in need of support. In both cases, however, the aid provided by tourists and the regeneration of local territories are mostly described as spontaneous processes without risks, incidents, or tensions:

“With the money collected by the association where my parents are members, we bought material, clothes, and even food, which we distributed to the villages and the schools.” (TS_14).

These experiences are oriented toward escaping and avoiding too many cultural mediators and mass tourists, still this approach is accompanied by strong activism in local communities. For tour operators, these tourists are a potential target, still they are also perceived as threatening because they pretend to do everything by themselves, with the risk of damaging local production systems and consumption and creating a power imbalance.

“Sometimes we have to cope with these travelers who present themselves as volunteers. We say that it is better to lose a little spontaneity but to rely on local tour operators, NGOs, who have been working in local communities for a long time and are more aware of how money is used, what are the right channels and the skills that they need to truly support the development of communities ethically to avoid risking doing worse.” (TO_06).

### The All-Inclusive Esthetics

This narrative reflects mainly ecotourism experiences characterized by an esthetic vocation and *slowness* orientation.

“I started because I liked snorkeling, then I was in contact with those from the WWF because I have always been one who likes animals, and I discovered this adventure field. At that point, my dad said, ‘Go,’ and he signed me up, and I went. Then, I found myself satisfied with their proposal, and my passion started from there. Initially, my interest was more naturalistic; then, I also approached the more ‘human’ side of contact with people.” (TS_01).

Unlike the first narrative—the “going native” myth—that shares a low-impact idea of sustainability, the contemplative and naturalistic aspect appears stronger here, together with the acceptance of mediators, such as local or international tour operators and guides often used for support or access to specific peripheral locations. The themes of green mobility and the pollution tourists’ presence entails are also strong in these narratives.

“The bicycle is a means that does not pollute. When traveling by other means, of course, we also have to compensate for the pollution, among other aspects. For example, we try to collect the plastic bottles that we give to everyone and that customers tend to throw away at the end of the trip.” (TO_05).

“Whenever possible, we also support the use of the train, and then we have a group of tour operators who prefer non-motorized travel: we also join the ‘alliance for soft mobility’ movement, which brings together all those who offer travel based on walking, riding bicycle or horse, sailing… the reuse of abandoned railways…” (TO_01).

In these narratives, tourists focus on themselves and their esthetic experience. The economic issue is not problematized, and saving or spending money do not appear to be of concern. Here too the theme of escaping from the mass is strong, while curiosity toward local cultural communities tends to be more marginal. Tourists want to enjoy best the relationship with the natural environment. Fatigue, as an integral part of an idea of the conquest of inaccessible and uncontaminated places, often accompanies the esthetic and contemplative dimensions. However, tour operators describe this fatigue as *constructed* and designed *ad hoc* according to tourists’ needs. It is therefore presented as an integral aspect of the services that tourists want. The myth of direct contact with nature and the local cultural environment is in the background compared to the high-quality and satisfactory travel experience. In this narrative, the consumer–tourist legitimizes fatigue but not necessarily uncertainty or risks.

“These trips are no more demanding and heavier than conventional ones. The trips we organize guarantee comfort equal to all the others. Fatigue can be calibrated and dosed as needed.” (TO_01).

### Intercultural Encounters

In this narrative, a sustainable tourist experience is a gradual approach to intercultural exchange moments, where tourists and local communities play an active role in seeking dialog and reciprocal understanding. Although tourists’ ethical vocation persists, it is accompanied by high awareness of the complexity of the interests and power dynamics that revolve around their travel experiences. Thus, in this narrative, tourists and tour operators reflect on the effects of their experience on the destinations’ economic, natural, and social ecosystems.

“Plunging into foreign worlds is complex. There are thousands of misunderstandings; even believing that you are a friend and welcomed in every place is naive. communication with other cultures is not so simple. One must not pretend that everything is easy. You should be satisfied with an intermediate level of involvement; you should limit yourself and your curiosity toward others; that’s how you can get in touch with local people. I remember that I am always a tourist; yes, I bring some money, and I am accepted for that too. But without being naive in this. Not everyone is inclined to meet others; it may be that in the other people, there is no curiosity and that the desire to distance one’s self from others prevails. You have to understand where you need to stop and where you are allowed to take some steps forward.” (TS_09).

The theme of getting in and out of the local communities is problematic due to potential conflict informing every intercultural exchange. A respectful curiosity about the encounter, recognized as a challenge, replaces the search for spontaneity and authenticity. Tourists themselves are also aware that they are sometimes the first *object* of exploration and even cultural manipulation by the locals.

“I was fascinated by my last trip to Iran. Being in a large group of visitors, the locals interviewed us. After the interview, they asked us what we thought of them. They specified that they were not violent people but pacifists. Traveling also opens the mind to different cultures and makes us understand how superficial our perspective is sometimes. They told each other that television depicts them as warmongers, but they are not like that. ‘We want to have peaceful relations with the rest of the world.’ Iraqi women are very keen to say that they made the revolution and protest every Wednesday because they want the freedom to decide whether to wear the veil.” (TS11).

In these travel experiences, the presence of intercultural mediators such as tour operators is legitimized because it allows better decoding of each other’s cultures and facilitates understanding by protecting each side against possible voluntary and involuntary offenses and threats. A mediation network—carried out by local and international gatekeepers—is fundamental for a respectful and responsible journey and allows for a meaningful encounter with the locals.

“We work with the Moroccan network to propose to young people to form meaningful encounters with local people. We have tourists who want to spend 10–15 days during which they try to live inside a village, they do a part of social work, together with a series of excursions with local guides.” (TO_06).

As can be read in the excerpt above, tourists’ experience has an active and committed implication, but always within the limits and the boundaries proposed by the intercultural mediators. The values of freedom and autonomy in traveling are partly sacrificed by using local and trusted operators, which guarantee that power and voice are given to local communities and that their development remains their responsibility. In describing these travel experiences, tourists’ economic concerns are made explicit. Here tourists are not necessarily looking for savings but critical consumption practices. Their journey is thus described as being a responsible consumer experience, in which travelers represent themselves as oriented to ensure that the production and purchase chain is guaranteed and local development is favored. The aim is to generate value for a common future.

“These tourists reflect on what can be generated and transformed into the places they visit because of their travel. They sometimes face ethical dilemmas regarding the degree of cultural contamination and transformation they cause and face themselves. Therefore, their contribution is weighted and reflected on rather than spontaneously given because they worry about what might remain or be transformed in the future.” (TO_05).

### Tourists and Tour Operators’ Positions: Tensions and Interlacements

As stated above, the four narratives do not represent four different types of tourists or tour operators. However, it can be said that tourists’ accounts have contributed most to narratives 1, 2, and 3, while narrative 4 (*intercultural encounters*) reflects mainly tour operators’ accounts. Tour operators mostly describe their role of mediators between tourists and local stakeholders (narratives 3 and 4); some of them also cite the *redemption travels* position (narrative 2), but only for distancing themselves from a kind of travel that, in their opinion, is too naïve and in some cases also harmful for the local communities. On the other hand, tourists have shown more representations of tourism sustainability; however, only two of their accounts contained elements contributing to the *intercultural encounter* narrative. This difference triggers tensions between diverse representations of who is perceived as legitimate and responsible for generating a sustainable experience.

On the one hand, tourists represent themselves as the main protagonists of the travel, by emphasizing their “direct” relation with the natural environment or with the local communities. They seem to perceive tour operators as “invisible” agents useful only to gain access to remote places or obtain travel/cultural information and advice. Tour operators, on the other hand, emphasize the importance of their role. They, however, differ in terms of how they characterize their role. In *all-inclusive esthetics*, the idea is that tour operators should organize everything and take all the responsibilities for creating a higher satisfactory travel experience. On the other hand, in the *intercultural encounter* narrative, they play an empowering and facilitating role for the tourists and the residents, valuing their differences and safeguarding the leading role of all the parties. Tour operators here show their effort to “set the ground” for the encounter by educating tourists and enabling the local communities to develop their services and sometimes to manage them. They also highlight the need for all the parties to be responsible and play their role in making the experience sustainable.

These aspects have several implications, which will be discussed in the following section.

## Discussion

This study’s first contribution is that tourists and tour operators describe different practices and experiences under the same label of sustainable tourism. These differences are rooted in diverse assumptions of sustainability and the value produced by a sustainable tourism experience, and they also have several implications for the perceived role of tourists and tour operators in this process.

In the first narrative, the sustainability *value* seems limited to the environmental *preservation* dimension, according to which tourists experience a complete immersion in the destination’s natural and cultural environment. Considering this idea of not disturbing and not corrupting the places/communities visited, the awareness that tourism is a process, which inevitably challenges consolidated balances that should be re-generated, is limited (Goodwin, 2011). The concept of sustainable tourism as a low-impact non-mediated experience seems to be consistent with an *ethnocentric* approach to sustainability, in which tourists and their interests, such as their need for freedom and their desire for discovering, appear to be the most “salient stakes” to be protected (Newton, 2002). This orientation, however, appears naive, as it ends up reinforcing a principle of separation between nature and culture/society, in which the tourist is seen as dominant, the local destination and its residents are viewed as passive, and the relationship between them is considered as “consumption” to avoid in favor of “preservation” (Caruana et al., 2014).

In the second narrative, the value generated by a sustainable tourism experience seems to be the product of a *transaction.* Tourists compensate for their visit by returning resources and helping the local communities. When providing help and care, power asymmetries between tourists and local communities do not seem to be questioned; a “responsible” but still *ethnocentric* view of sustainability appears to be reinforced instead (Dangi and Jamal, 2016). Tourism is seen as an impactful experience that facilitates repaying environmental and cultural “consumption” by providing social help and economic resources. This idea is framed within a paternalistic view of the relationship between tourists and residents who do not get a chance to decide what is sustainable for them (Castiglioni et al., 2019). Here again the social, environmental, and economic resources exchanged between tourists and residents are considered separate and interchangeable (Allen et al., 2015, 2017). Several criticalities can be noticed in this view, such as the presence of an idealized perspective in tourist–residents dialog, which ignores potential misunderstandings, manipulations, and inequalities (Loìpez-Saìnchez and Pulido-Fernaìndez, 2016).

In the third narrative, the value produced in a sustainable tourism experience seems mostly *esthetic*, focusing on tourists and their search for exclusivity and naturalness, which are considered indicators of good quality service. Unlike in the first two narratives, the mediating effects of tour operators are recognized and legitimized. Accordingly, tour operators are asked to guarantee a high-quality travel experience, which reduces the relationship with and effects on the local communities. Here the concept of sustainable tourism seems to be consistent with an *ecocentric* approach to sustainability, in which the natural environment appears to be the most “salient stakeholder” to be safeguarded (Newton, 2002). However, this ecocentric orientation appears to be idealized, as it reinforces a principle of separation between nature and culture/society in which only the “human” is seen as powerful. We agree with Allen et al. (2017), who recognize several criticalities in this view of nature, which is seen as “fixed” and “perfect,” while the flux and instability of ecosystems are ignored. This idealized ecocentrism is also consistent with the idea of clear boundaries between humans and nature, overlooking socially constructed ideas about nature.

In the fourth narrative, the value produced by sustainable tourism is essentially seen as *co-generated*. It refers to creating new relational and learning opportunities for all the parties involved. The relationship between tourists and tour operators and between tourists and local communities is a core value here. Tour operators are asked to play the role of cultural mediators, handling power asymmetries and taking on the responsibility for the ethics of the exchange. In this narrative, the idea of sustainability is legitimized as *embeddedness* (Allen et al., 2015, 2017), that is, as an experience in which human, natural, cultural, and economic dimensions are intertwined and considered from a long-term perspective. Here sustainability is viewed as a never-ending and open “journey” generated from multi-stakeholder dialog, where stakeholders are not only social human but also non-human agents (e.g., the natural environment). Furthermore, such dialog is reflexively addressed by considering its dilemmas and limits. In this sense, for instance, both tourists and tour operators are able to problematize tensions between the economic viability of the travel vs. the value of critical consumption practice, between the desire to discover vs. the limits imposed by mediators, or between the search for authenticity vs. the risks of misunderstandings.

The study’s second contribution is that each narrative has different educational and communication implications for enhancing awareness of tourism sustainability. In the first and the second narratives, promoting cultural development toward a more aware and integrated view of sustainability requires a process of de-idealization of some preconceived notions, such as the implicit ethnocentrism of this practice, the illusion that tourism might not influence the destination equilibrium, the ideal of a harmonic encounter with other cultures, and the overlooking of the intercultural competencies needed to facilitate a good-quality and sustainable tourism experience. Another promising lever could be the development of new storytelling around sustainable tourism, which should trigger the idea of sustainability as co-generation of value rather than as (low) consumption, through giving voice not only to tourists’ expectations and values but also to local communities’ expectations and needs. In this regard, tour operators and public policymakers should raise more awareness of the actual complexity of crafting sustainable tourism and also warn tourists against the risk of seeing it as a “do it yourself” practice. In the third and the fourth narratives, it seems possible to appeal to the reciprocal trust shown by tourists and tour operators to develop a higher awareness of and a critical reflexive stance toward their respective roles and responsibilities to reinforce their alliance. In both narratives, tour operators are legitimated as necessary gatekeepers and guarantors of the sustainability of the experience. However, in the third narrative, the relationship between tourists and tour operators resembles a conventional arrangement between a provider and a consumer. The former offers exclusive service, and the latter renumerates for a high-quality natural and cultural experience. Only in the fourth narrative did tourists and tour operators seem to question their taken-for-granted assumptions and elaborate on the limits and tensions of every intercultural encounter. Since the fourth approach is the one that best interprets a critical and holistic view of sustainability, although it reflects only a few participants’ accounts, it could be said that much more effort is needed to further spread this perspective among tourists and tour operators. Tourists need to become increasingly aware of the physiological misunderstandings, cultural conflicts, risks, and uncertainties of “sustainable” traveling. Tour operators need to welcome this nuanced view of sustainability and incorporate eco-tourism and responsible and community-based tourism into a wider and more integrated approach. They also need to develop more competencies in the area of intercultural mediation, community empowerment, and tourists’ education.

From a communication perspective, new storytelling should center on sustainable tourism as a process where many different actors are connected to co-generate new social, economic, and environmental wealth. Such storytelling should emphasize that all stakeholders must be open to “be surprised” by the encounter and revise each others’ implicit beliefs and cultural preconceptions. In this way, a higher critical awareness of each actor and the consequent opportunities for learning and improving could arise.

## Research Study Limitations

The research has several limitations. First, the perspective of residents has not been considered. This has limited the possibility of investigating their sustainability experience and their perceived relationship with tourists and tour operators. Further research studies are therefore needed to address this gap. Second, the study’s qualitative and interpretive nature does not allow us to describe the distribution of tourists and tour operators across the four narratives. To overcome this limitation, other studies should describe in more detail the tourists’ and the tour operators’ cultural and socio-demographic characteristics. Finally, the COVID-19 crisis is having a deep effect on the tourism sector and its sustainability. New studies should be developed to explore the potential increase in the awareness of sustainability due to this actual and relevant phenomenon and its implications for the lives of tour operators, tourists, and residents and for sustainable value production.

## Conclusion and Implications for Theory and Practice

Sustainable tourism is a considerably debated issue due to a growing awareness of the environmental, social, and cultural effects of tourism activity. The present study discusses the lack of a widespread and consolidated holistic approach to sustainable tourism. The concept of sustainability remains ambiguous, nuanced, and partial. The article argues that this happens because an integrated and “embedded” view of sustainability—as identified in only one of the four narratives of the study—requires a radical transformation of the ontological and epistemological assumptions regarding the relations between the human and the natural environment, where nature is considered a proper stakeholder and where all the parties involved are given equal power and agency.

The study has some implications for theory and practice. From a theoretical point of view, the article suggests the importance of avoiding ethnocentric or idealized ecocentric perspectives on sustainability and adopting a more critical and holistic framework. This approach might also allow a new operationalization of sustainable tourism, where social, economic, and environmental indicators can be grounded into concrete repertoires of experiences. From a practical point of view, the article has implications for tourists’ and tour operators’ cultural development. New educational approaches are needed to change tourists’ and operators’ idealized and contemplative view of tourism—where tourists are seen as spectators or consumers of the natural/cultural/social and economic resources of a destination—to a higher awareness of their power and responsibilities in co-generating a sustainable tourism experience. Furthermore, critical reflexivity of tourists and tour operators should be promoted to improve their understanding of the various interests in every tourism experience and to allow them to reflect on the social, economic, and environmental consequences of every action. Sustainable tourism communication should also be improved by designing new storytelling where the social, economic, and environmental standards of a sustainable destination and the stakeholders’ involvement in co-generating sustainable value can be highlighted.

## Data Availability Statement

The raw data supporting the conclusions of this article will be made available by the authors, without undue reservation.

## Ethics Statement

Ethical review and approval was not required for the study on human participants in accordance with the local legislation and institutional requirements. The patients/participants provided their written informed consent to participate in this study.

## Author Contributions

All authors listed have made a substantial, direct and intellectual contribution to the work, and approved it for publication.

## Conflict of Interest

The authors declare that the research was conducted in the absence of any commercial or financial relationships that could be construed as a potential conflict of interest.
